# A pill as a quick solution: association between painkiller intake, empathy, and prosocial behavior

**DOI:** 10.1038/s41598-023-45267-0

**Published:** 2023-10-26

**Authors:** Magdalena Banwinkler, Markus Rütgen, Claus Lamm, Helena Hartmann

**Affiliations:** 1 Department of Cognition, Emotion, and Methods in Psychology, Faculty of Psychology, University of Vienna, Vienna, Austria; 2 Department of Nuclear Medicine, Faculty of Medicine and University Hospital Cologne Germany, University of Cologne, Cologne, Germany; 3 Department of Clinical Neuroscience, Karolinska Institutet, Stockholm, Sweden; 4 Clinical Neurosciences, Department for Neurology and Center for Translational and Behavioral Neuroscience, University Hospital Essen, Essen, Germany

**Keywords:** Empathy, Social behaviour

## Abstract

Previous research has demonstrated a link between the administration of analgesic drugs and the reduction of empathy levels in humans. This apparent blunting effect of pain medication has been explained through shared neural mechanisms for the first-hand and the empathic experience of pain (simulation theory). Considering that analgesics are among the most consumed drugs in the world and the ability to empathize with others is fundamental to human social interactions, the aim of the present study was to investigate whether the typical day-to-day analgesic consumption rate in Austria and Germany is associated with a reduction in empathy and prosocial behavior. We therefore collected self-reports of analgesic consumption behavior as well as empathy for pain and prosocial behavior measures in an online survey (*n* = 940). Analyses revealed no significant association between the analgesic intake frequency and measures of empathy or prosocial behavior. However, liberal intake of analgesics (i.e. mind-set of “a pill is a quick solution”) was linked to lower empathic concern and helping behavior, which may hint towards a negative effect in people who take pain medication for non-pain related issues or episodes of low pain. Nevertheless, further research is needed to investigate the effects of analgesic drugs in high frequency users.

## Introduction

Pain is a central part of human daily life and constitutes one of medicines’ oldest problems. Medication is the most commonly used method for pain management and these days, our society is seeing a steady increase in the use of analgesics, which is fueled by trends such as increased self-medication and easy access to over-the-counter (OTC) drugs^1–4^. Today, analgesics are among the most consumed drugs in the world and millions of people are estimated to use OTC painkillers on a day-to-day basis^5,6^. This may have effects that go beyond the mere reduction of pain as alterations in psychological and social aspects (e.g. blunted reactions to emotionally arousing images and reduced reports of social pain) have been reported in association with the intake of analgesics^7,8^.

One of the most frequently and widely used OTC analgesics is paracetamol (also known as acetaminophen or N-acetyl-*p-*aminophenol). Paracetamol is a centrally acting analgesic and antipyretic (fever reducing) drug, which is commonly used for the treatment of mild-to-moderate pain, such as headaches, sprains, and back aches^9–11^ as well as for the treatment of chronic pain such as osteoarthritis^12^. Paracetamols’ classification as adequately safe, gained it OTC status which contributed to its widespread usage^11^. Prevalence estimates suggest that in the US, paracetamol is taken by 23% of the adult population each week^13^ and recent studies have revealed a similar pattern in Europe. In France paracetamol is the most widely prescribed drug with 51% of the population above 15 years being dispensed paracetamol at least once in a year^14^; in Germany one in five adults between the age of 18 and 79 years uses analgesics in a given week, whereby paracetamol is the third most used analgesic^15^; and a Swedish population-based study found that 70.5% of participants reported use of paracetamol in the last three months^16^.

These high numbers inevitably raise questions about potential consequences of paracetamol intake. Besides the very well documented and desired analgesic and antipyretic effects of paracetamol, the painkiller demonstrates more widespread effects, exerting its influence also on psychological and social aspects. For instance, paracetamol has been shown to reduce self-reports of hurt feelings and to decrease neural responses to social rejection^7^, to reduce trust and feelings of social integration^17^, to reduce the compensatory response to violations of expectations^18^, and to dampen affective reactivity for both emotionally negative and positive images^8^. In accordance with this assumed blunting effect of evaluative and emotional processing, the results of Keaveney et al.^19^ indicate that paracetamol can also cause increased risk taking by reducing risk perception.

One particularly interesting and potentially far-reaching effect of paracetamol is, that it can also impact empathy levels, as it has been shown that paracetamol reduces the capacity to empathize with another person’s painful experiences. Mischkowski et al.^20^ demonstrated that the administration of a single paracetamol dose lead to a reduction in empathy levels when reading scenarios about another person experiencing physical and social pain, when whitnessing an actual incident of social pain, as well as when imagining another person receiving painful noise blasts. While these findings seem to suggest that paracetamol specifically reduces empathy for pain, a follow-up study found that positive empathy, e.g., the ability to share feelings of happiness with another person, was reduced as well, following the administration of paracetamol^21^.

One explanation for this extended effect of paracetamol is provided by simulation theories, which state that empathizing with another person’s pain and feeling pain recruit similar neural mechanisms^22^. In fact, similar brain areas seem to be active while seeing someone else in pain and while experiencing first-hand pain^23–25^. Mechanistic support for these “shared representations” is provided by placebo analgesia studies, which demonstrated decreased empathy for pain as well as decreased engagement of brain areas previously associated with pain and empathy for pain (anterior midcingulate cortex, anterior insula) after the administration of a placebo pill presented as a ‘potent painkiller’^26,27^. Important evidence in favor of this assumption also stems from an EEG study^28^. After placebo analgesia induction, the administration of the opioid receptor antagonist naltrexone (which is known to reverse the previously induced analgesic effect) led to increased self-reports of first-hand pain as well as empathy for pain. Importantly, P2 (an event-related potential component reliably associated with first-hand pain) amplitudes were also increased during both, first-hand pain and empathy for pain. Thereby, this study demonstrated that the direct manipulation of neurochemical processes of a pain-regulating system has the potential to affect self-experienced as well as empathic pain. Furthermore, recent evidence indicates that placebo analgesia has domain general effects, affecting not only empathy for pain but also empathy for unpleasant touch. However, only in the in the pain context this effect was reversed by the administration of an opioid antagonist, supporting the role of a specific shared neurochemical system for pain and empathy for pain^25^.

Paracetamol absorption via the oral route occurs rapidly and peak plasma concentrations are attained 0.5–1.5 h after intake. A plasma half-life of about 2–2.5 h has been reported and 90–100% is washed out after a time period of 24 hours^29,30^. Considering the view of the pharmacokinetics of paracetamol, one would not expect long-lasting effects on psychological processes, or social cognition and behavior. However, opioid intake for example has been associated with sustained deficits in socio-cognitive functioning, as it was shown that regular opioid intake lead to decreased emotion recognition in measures of cognitive empathy^31^ as well as decreased empathic concern^32^. Moreover, trait empathy measures rely on questions where people report what they commonly do or how they react to the suffering and emotions of others. This naturally relies on introspection to report one’s past, self-experienced empathy, and thus indirectly on the episodic memory of those experiences^33^. From previous studies, it is known that state-induced lowering of pain sensitivity can reduce empathy^20,26,27^. If a person takes pain medication on a regular basis and over extended periods of time it thus seems plausible to assume that the painkiller intake might reduce the amount of empathic events and experiences a person has. Following this rationale we would even expect to be able to derive graded responses: the higher or more frequent the analgesia intake, the stronger the negative effects. Thus, it is conceivable that paracetamol by means of its short term effects on self- and other-related emotional processing may lead to long-lasting effects, and their self-report, especially in heavy users, although the exact pharmacological mechanism of action of Paracetamol is still unclear^34^.

The ability to empathize with others is a fundamental component of our daily social life, contributing to adequate social interactions^35^. The increasing consumption rate of painkillers in combination with the striking potential of a single dose of paracetamol to reduce empathy, raises a very crucial question: What effect does this common (i.e. the typical day-to-day) analgesics consumption behavior exert on individual empathy levels and thus on our daily interpersonal interactions? Opioid users demonstrate impairments in recognizing emotions and others feelings, pointing out the potential implications of analgesic consumption on social functioning^31^. Paracetamol has been found to reduce empathy in young adults in experimental setting^20,21^. However, it is still unclear if these differential effects on empathy are short-term and only detectable under ideal experimental conditions or in certain patient groups, or if these effects persist over prolonged time periods (thus, affecting trait empathy as seen in opioid users) and are large enough to be detectable in doses which are commonly consumed on a day-to-day basis.

Furthermore, research indicates that empathy plays an essential role in the regulation of prosocial behavior^36^. Both affect sharing and mentalizing are considered to be important motivational factors for the engagement in prosocial behavior, e.g. by enabling a better understanding of another person’s needs^37,38^. Relating prosocial behavioral tendencies to the intake of analgesics, a recent study that employed a rat-model showed reduced prosocial-like behavior after the administration of paracetamol^39^. Furthermore, a recent preprint showed that down-regulated pain sensitivity via placebo analgesia transferred to prosocial behavior, measured as choosing to put in physical effort to prevent painful shocks for another person^40^. This study found that individuals under the influence of placebo analgesia not only chose to help less often, but also demonstrated slowed reaction times when choosing to help or not, as well as less energy even after having chosen to help, in comparison to individuals with typical pain sensitivity. Considering this finding in combination with the close connection between empathy and prosocial actions, we extended our research question, focusing not only on empathy but on prosocial behavior as well.

In this context, we conducted an online study to examine whether the amount of analgesics that is consumed on a day-to-day basis is associated with as a reduction in empathy and prosocial behavior. We aimed to follow up the experimental research in this field by investigating if participants with a high frequency of analgesia intake or the tendency to quickly reach for analgesic drugs demonstrate significantly reduced trait empathy and prosocial behavior.

## Materials and methods

The present study was conducted according to the principles expressed in the 2013 Declaration of Helsinki. All procedures were approved by the local ethics committee of the University of Vienna beforehand (application number 00412). Participants were informed about the nature of the online questionnaire on the landing page and provided informed consent by clicking a button to proceed to the next page.

### Study design

An online survey which was primarily aimed at young Austrian and German adults but included all individuals above the age of 18 years was conducted to assess analgesic drug intake frequency, how liberal analgesic drugs are consumed, as well as trait measures of empathy and prosocial behavior. Participants completed all questions online via SoSciSurvey^41^. The survey took around 15–20 min to complete. Subjects did not receive any financial compensation. The study was not formally preregistered.

### Participants

In total, 1097 participants completed the survey. A subset of 121 participants were taken from a behavioral study conducted at the Faculty of Psychology of the University of Vienna^40^. Subjects who reported the use of neurological or illicit psychoactive drugs (*n* = 148), as well as subjects with invalid responses (e.g. wrong age or intentionally wrong answers; *n* = 9) were excluded from the analysis. The final sample included 940 participants (752 women, 188 men) aged between 18 and 92 (*M*_age_ = 26.39, *SD* = 8). Forty-four percent of the sample had a bachelor’s degree or higher education. For an overview of participant characteristics, see Table 1.

**Table 1:** Participant characteristics.

	*M*	SD	*n*
Identified as female/male	-	-	752/188
Age (years)	26.39	8.00	940
Analgesic drug intake (times per week)	0.55	0.76	940
Paracetamol intake (times per week)	0.22	0.42	940
Empathy for Pain Scale			
Affective distress	2.68	0.75	819
Empathic concern	3.74	0.67	819
Vicarious pain	2.10	0.92	819
Helping Attitudes Scale	78.61	9.21	940
Liberal analgesic use	28.85	6.37	940

### Measures

Trait empathy was self-assessed by participants filling in the empathy for pain scale^42^ (EPS). The EPS measures empathic reactions to seeing another individual in pain across four different scenarios and consists of three subscales: Affective Distress, Empathic Concern and Vicarious Pain. The EPS was not completed by participants who were part of the behavioral experiment (*n* = 121), resulting in a lower sample size.

Prosocial and helping tendencies were measured via the helping attitudes scale^43^ (HAS), which was designed to measure beliefs, feelings, and behaviors related to helping others.

To assess the use of analgesic drugs, participants were asked a set of questions regarding their daily analgesic drug consumption behavior. This included questions about the duration and frequency of analgesic intake. As the previous study by Mischkowski et al.^20^ focused on effects of paracetamol on empathy, participants were additionally asked to specify if they consumed a paracetamol-containing drug within the last three months. To avoid wrong answers due to a lack of knowledge regarding the active ingredient, participants were provided a list with the brand names of all OTC-paracetamol-containing analgesics available in Austrian pharmacies. For each analgesic, participants had to report if they did or did not consume a medication of this brand in the last three months.

In order to assess how hesitant vs. fast participants resort to analgesic drugs, the liberal intake of analgesics was measured with a self-designed scale of 16 questions (see Table 2). Example items are: “I take pain medication already when experiencing mild pain” and “I only take pain medication if it cannot be avoided”. Each item was rated on a 4-point Likert scale, ranging from *strongly disagree* to *strongly agree*. A total score was computed as sum of the individual item ratings, with the scores of items 4, 6–9, 11 and 14 being reversed.

**Table 2:** Items of the self-designed liberal-analgesic-use scale.

Nr	Original in German	English translation
1	Ich nehme, ohne groß darüber nachzudenken, Schmerzmittel ein	I take pain medication without putting much thought into it
2	Ich nehme Schmerzmittel bereits bei geringen Schmerzen	I take pain medication already when experiencing mild pain
3	Ich nehme bei Kleinigkeiten schnell mal eine Tablette	To treat small issues, I quickly take pain medication
4	Ich finde Medikamente unnötig	I believe pain medication is unnecessary (reverse coded)
5	Ich nehme leichtfertig Schmerzmittel zu mir	I take pain medication liberally
6	Ich lese mir die Packungsbeilage durch	I read the package inserts of drugs (reversed)
7	Ich nehme Schmerzmittel nur wenn es unbedingt notwendig ist	I only take pain medication when it is absolutely necessary (reverse coded)
8	Ich nehme Schmerzmittel nur wenn sie von einem Arzt verschrieben worden sind	I only take pain medication when prescribed by a physician (reverse coded)
9	Ich informiere mich über ein Medikament bevor ich es einnehme	I inform myself about a drug before taking it (reversed)
10	Schmerzmittel stellen eine schnelle Lösung für meine Probleme dar	Pain medication is a fast solution to my problems
11	Mit der Einnahme von Schmerzmitteln sind Risiken verbunden	The use of pain medication is associated with risks (reverse coded)
12	Es ist komplett unbedenklich Schmerzmittel einzunehmen	It is completely harmless to take pain medication
13	Ich nehme präventiv Schmerzmittel ein (z.B. vor dem Sport, während der Periode, etc.)	I take pain medication preventatively (e.g. prior to doing sports, during menstruation)
14	Ich nehme Medikamente nur, wenn es sich nicht vermeiden lässt	I only take pain medication if it cannot be avoided (reverse coded)
15	Ich nehme Schmerzmittel gegen den "Kater" (z.B. ausgelöst durch Alkohol oder Drogenkonsum)	I take pain medication to treat a hangover (caused by alcohol or drug consumption)
16	Ich denke, dass mein Medikamentengebrauch (z.B. Häufigkeit der Anwendung, Einnahmegründe) problematisch ist	I believe my medication consumption behavior is problematic (e.g. intake frequency, reason for intake)

### Statistical analysis

For statistical analyses RStudio version 4.0.3 was used. Normality of the variables was checked with the Shapiro–Wilk test. Due to non-normal distribution, non-parametric Spearman rank correlations (*r*_s_) were calculated to investigate the relationship between the intake frequency of analgesics in the past 3 months and (a) the EPS as well as (b) the HAS. These two statistical analyses were conducted twice, one time including all types of analgesic drugs and one time explicitly focusing on paracetamol. We additionally checked the validity of our newly designed liberal-analgesic-use scale by correlating it with analgesic intake frequency, once for all analgesics and once specifically for paracetamol. The relationships between the liberal analgesic use and the intake frequency of analgesics, the intake frequency of paracetamol as well as the EPS and HAS, were also analyzed using Spearman rank correlations. Outliers were dealt with by the means of winsorization. Accordingly, all values lower than the 5%-quantile were replaced by the 5%-quantile value and all values larger than the 95%-quantile were replaced by 95%-quantile value. This criterion was applied to 45 and 36 values of the paracetamol and analgesic data respectively. Please note that this winsorization process did not change the overall pattern of the findings, as an analysis without winsorization led to largely identical results (see Supplement). The statistical significance level was set to *p* < 0.05, with Bonferroni thresholds for multiple comparison corrections indicated where applicable.

## Results

**Figure 1:**
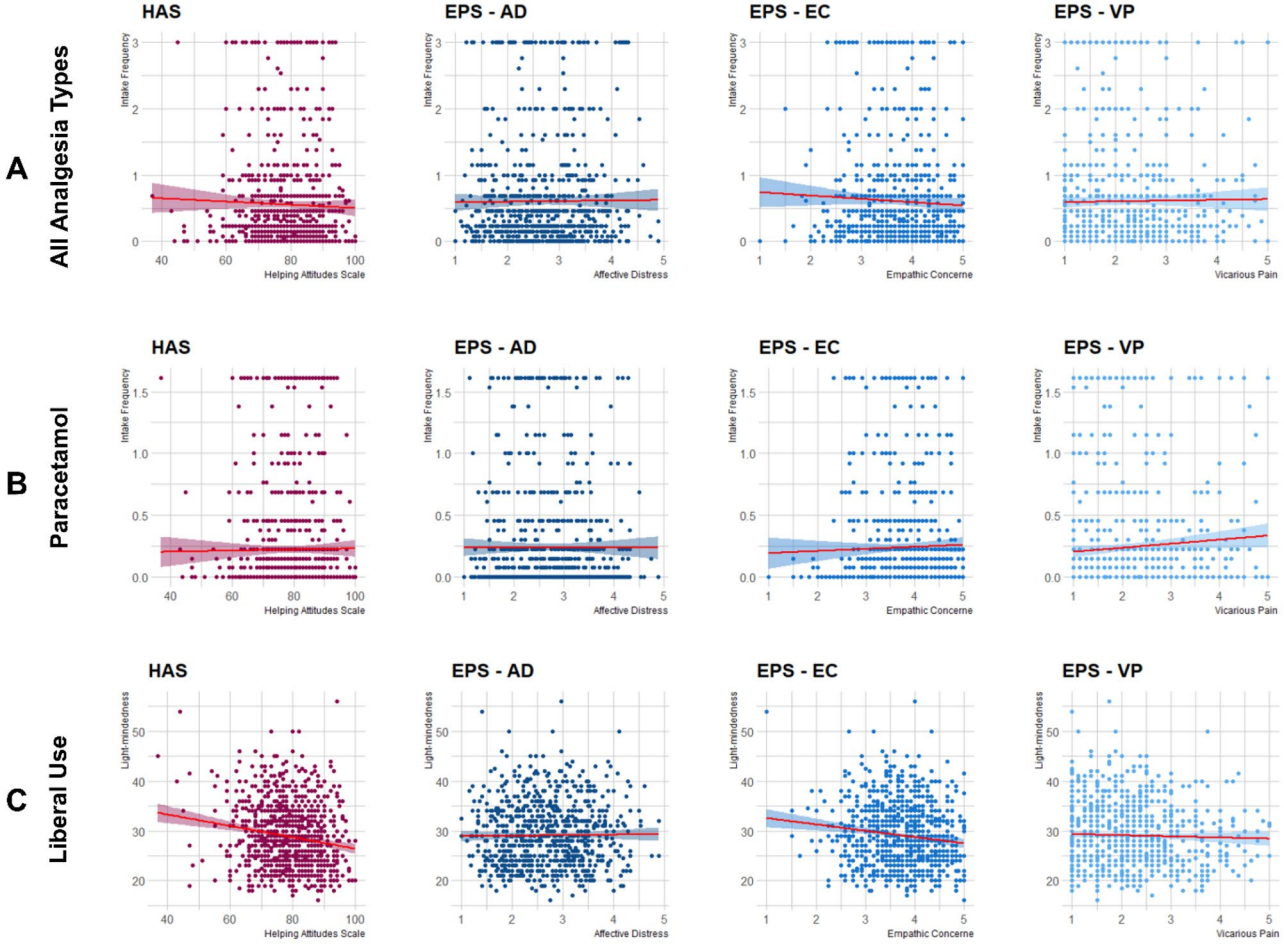
Spearman correlations between analgesia intake in the past three months and prosocial behavior measures with the helping attitudes scale (HAS) as well as the subscales affective distress (AD), empathic concern (EC and vicarious pain (VP) of the Empathy for Pain Scale (EPS). Sub-figures are separated by (**A**) all kinds of analgesia intake, (**B**) only paracetamol intake and (**C**) liberal use of analgesic drugs.

Seventy-seven % of participants (*n* = 722) reported at least one analgesic intake and 45% (*n* = 422) at least one paracetamol intake in the past three months. Mean analgesic intake per week was 0.55 times and mean paracetamol intake per week was 0.22 times. There was no significant relationship between the frequency of analgesic intake and trait helping behavior: *r*_s_ =  − 0.01, *p* = *0*.856. There was also no significant relationship between the frequency of analgesic intake and the level of self-ascribed affective distress (*r*_s_ =  − 0.01, *p* = 0.739), empathic concern (*r*_s_ =  *− 0.01*, *p* = 0.688), and vicarious pain (*r*_s_ = 0.01, *p* = 0.873; see Fig. 1A). The paracetamol intake frequency was also not significantly related to helping behavior (*r*_s_ = 0.03, *p* = 0.308), affective distress (*r*_s_ = 0.02, *p* = 0.645), empathic concern (*r*_s_ = *0.*06, *p* = 0*.*107), or vicarious pain (*r*_s_ = 0.07, *p* = *0.*058; see Fig. 1B). Together, these analyses do not show evidence for a relationship between analgesics, or specifically paracetamol, intake in the last three months, and participants’ trait empathy for pain and prosocial behavior.

The liberal-analgesic-use scale demonstrated a significant positive correlation with the frequency of analgesic intake (*r*_s_ = 0.44, *p* < 0.001) as well as paracetamol intake (*r*_s_ = 0.27, *p* < 0.001). Interestingly, the liberal use of analgesics was significantly negatively correlated both with helping behavior (*r*_s_ =  − 0.16, p < 0.001) and empathic concern (*r*_s_ =  − 0.12, *p* = 0.001), whereby higher subjective liberal use, i.e., faster resort to analgesic drugs, was associated to lower prosociality and lower empathic concern (Bonferroni-adjusted threshold of *p* < 0.0125). However, the liberal use was not significantly correlated with affective distress (*r*_s_ = 0.02, *p* = 0.665), and vicarious pain (*r*_s_ =  − 0.02, *p* = 0.624; see Fig. 1C). Additionally, in an exploratory post hoc analysis, we investigated a potential moderation effect of liberal use on the relationship between paracetamol intake frequency and the EPS as well as the HAS. The results revealed a significant interaction of liberal use and paracetamol intake frequency on helping behavior, *F*(3, 936) = 11.36, *p* = 0.023, whereby lower liberal use was associated with a positive correlation between paracetamol intake frequency and helping behavior. We did not find such an interaction in any EPS subscale (all *p*’s > 0.134).

## Discussion

The goal of the present study was to explore the effect of the common analgesic consumption rate in a large online-sample of young adults on trait empathy for pain and prosocial behavior. Following the work from Mischkowski et al.^20^, we hypothesized that a high analgesic intake frequency would be associated with lower levels of self-reported empathy and prosocial behavior, and vice versa. Besides the effect of analgesic intake in general, we were specifically interested in the effect of paracetamol. Contrary to our expectations, the intake frequency of analgesic was not related to lower empathy or prosocial behavior, nor was the intake frequency of paracetamol.

We did not observe a significant relationship between analgesic intake and empathy for pain or prosocial behavior measures, suggesting that the amount of painkillers which is commonly consumed on a day-to-day basis is not associated with a blunting effect of these aspects of social cognition. Accordingly, also no pain-specific effect of analgesic intake was observed. These findings do not appear to corroborate previous research in this area which has demonstrated reduced state-related empathy and prosocial-like behavior following the administration of paracetamol^20,39^.

However, there are several possible explanations for this apparent lack of correlation. First and foremost, the majority of participants in our sample were (a) young adults and (b) reported a relatively low analgesic intake frequency, with an average of one intake every two weeks. Rates of paracetamol intake were even lower. This limited age range, and the low consumption rate could have overcast a potentially existing blunting effect of paracetamol on empathy and prosocial behavior. Furthermore, different types of pain medication have different mechanisms of action, thus potential effects of paracetamol or other single painkillers might have been hidden because of the combined analysis approach. Thus, our findings do not oppose a possible effect but rather suggest that the rate of analgesic consumption which is observed in a sample of mainly young adults is not associated with a detectable blunting effect. Our findings should therefore be replicated in a sample with a larger age range, possibly focusing on participants who report a higher analgesics intake.

Another important point is the distinction between changes in state vs. trait empathy. To our knowledge most previous studies have focused on the acute effects of paracetamol administration on state empathy, which relates to context-specific empathy^44^. It is important to acknowledge, that it still remains unclear whether the consumption of paracetamol leads to a short-term alteration in state empathy and if the repeated intake over an extended time period can additionally lead to a reduction of the more stable trait empathy as seen in chronic opioid users^31^. Under the assumption that paracetamol is indeed affecting trait empathy levels of individuals, the lack of negative correlations between the intake frequency of the past three months and trait empathy measures could be based on the lack of high frequency users. Unfortunately, our sample did not include enough high-frequency users to warrant a subgroup analysis. Therefore, this interpretation should be the focus of future studies.

Another aspect, which should not be left unnoted while interpreting the present findings, is the effect of contextual factors. People who take painkillers, such as the participants in our study, have a certain motive to do so (i.e. to alleviate pain). The presence or absence of this motive also draws a clear line between studies which investigate healthy controls on pain medication in a lab setting versus studies which assess effects in people who take pain medication in real life. There are lines of research, which suggest that pain by itself may also reduce responsiveness to other people’s pain^45,46^. This could in fact lead to higher empathy levels on medication compared to off medication in individuals who experience high levels of pain. Considering the present findings under this assumption, analgesia intake would not be associated with observable negative effects on empathy or prosocial behavior. We did not actively measure the pain level or suffering of our participant; however, this could be an important variable for future studies.

The main strength of our study is that unlike previous studies, we investigated the effect of the actual common day-to-day paracetamol consumption rate, not the acute effect of one high paracetamol dose. In this regard our results relate more closely to the real-world setting, as we captured the actual consumption behavior. Our sample mainly consists of young Austrian and German adults, which may limit the generalizability to other populations. However, there is research which suggests that mere convenience samples can provide useful estimates^47^. Furthermore, we did not assess the amount of the active pharmaceutical ingredient in the pain medication, that was taken by each participant during the last three months. Future studies should therefore focus on longitudinal data collection procedures and work, for example, with medication diaries in order to have more complete data on medication use and exact dosage (e.g. how many milligrams of an active ingredient were taken how often). The present analyses were based on the number of doses, thus the drug intake frequency.

In order to further enhance and refine the assessment of the analgesic consumption behavior, a measure of how liberal analgesics are used was introduced as a proxy for the actual consumption behavior which is not restricted to the past three months. The results that emerged from this second analysis raise some interesting questions. Participants with a liberal analgesic use, which represents a faster resort to analgesic drugs, reported lower prosociality and empathic concern. In other words, people with an analgesic consumption behavior that is characterized by a mind-set of “a pill is a quick solution” show deceased prosocial and empathy for pain levels. One possible explanation for this result could be that this type of attitude and corresponding consumption behavior could in the long run have led to the observed decreased scores. This explanation would be in line with the shared representations account of empathy that posits that we use our own pain processing system to simulate, and subsequently empathize with, the pain of others^48,49^. In this context, individuals who have a higher tendency to avoid dealing with their own pain by reaching for pain medication faster, might also avoid stronger engagement with the pain of others or might have more issues coping with painful experiences in general. Importantly, this view matches with our results, as our empathy questionnaire specifically measured empathy in different painful experiences, such as pain resulting from witnessing surgery, assault, or accidental injury. Furthermore, following up on the above mentioned point regarding the potential negative effects of pain, one would expect to see the most pronounced detrimental effects of analgesia use on empathy and prosocial behavior particularly under circumstances in which people use analgesics for non-pain related issues or episodes of low pain. Corroborating this assumption, we did find lower prosociality and empathic concern in liberal analgesia users. Interestingly we found a positive association between paracetamol intake and helping behavior in non-liberal analgesia users, in an exploratory post hoc analysis. This result hints at the hypothesis that individuals who take medication for a reason (e.g. high pain) might demonstrate higher empathy or prosociality on medication. Additionally, we observed an association between a more liberal analgesia use and increased analgesia as well as paracetamol intake, which demonstrated the validity of our new scale and its relationship to actual analgesia use. However, we acknowledge that this scale is exploratory and that additional work needs to be carried out by independent researchers in order to establish its validity.

The present findings point toward promising future research directions and due to their novelty, they need replication and extension. There are various interesting options how the impact of paracetamol consumption on psychological and social aspects could be further examined. For instance, it would be desirable to replicate this study using different study populations, preferably including high-paracetamol-consumers, in order to examine if these individuals are affected by a potential blunting effect. Additionally, it is also worth to investigate the stability or variability of trait empathy, due to long-term paracetamol intake. This research questions needs a more directed approach, than was employed in the present study. This could be accomplished by the assessment of various trait measures pre and post of a paracetamol administration period.

## Conclusion

The main goal of this study was to deepen our understanding of the relationship between the common day-to-day analgesics use and empathy as well as prosocial behavior. In a sample of young Austrian and German adults, high analgesic intake frequency was not associated with lower empathy and prosocial behavior. However, the liberal intake of analgesics was linked to lower empathic concern and helping behavior, which may hint towards a negative effect in people who take pain medication for non-pain related issues or episodes of low pain.

### Supplementary Information

Supplementary Information.

## Data Availability

The datasets and analysis code supporting this article have been uploaded on the Open Science Framework (https://osf.io/dzs85/). All questions of the liberal-analgesic-use scale created for this study are reported in Table 2.
