# Supplementary material for: A pill as a quick solution: association between painkiller intake, empathy, and prosocial behavior
Source: Sci Rep. 2023 Oct 26;13:18320. doi: 10.1038/s41598-023-45267-0 (PMC10603176; doi:10.1038/s41598-023-45267-0)

**Supplement**

Results without winsorization of the *frequency of analgesic intake* and *frequency of paracetamol intake* variables are listed below:

There was no significant relationship between the frequency of analgesic intake and trait helping behavior: *r_s_* = -.01, *p* = .861. There was also no significant relationship between the frequency of analgesic intake and the level of self-ascribed affective distress (*r_s_* = -.01, *p* = .732), empathic concern (*r_s_* = -.01, *p* = .682), or vicarious pain (*r_s_* = .01, p = .876).

The paracetamol intake frequency was not significantly related to helping behavior (*r_s_* = .03, *p* = .316), affective distress (*r_s_* = .02, *p* = .647), empathic concern (*r_s_* = .06, *p* = .108), or vicarious pain (*r_s_* = .07, *p* = .057).

The liberal-analgesic-use scale demonstrated a significant positive correlation with the frequency of analgesic intake (*r_s_* = .44, *p* < .001) as well as paracetamol intake (*r_s_* = .27, *p* < .001).

Below we display the data before (uncorrected) and after (corrected) winsorization.


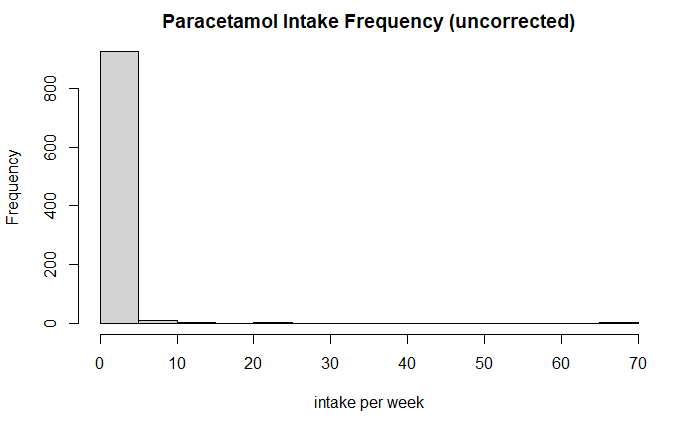

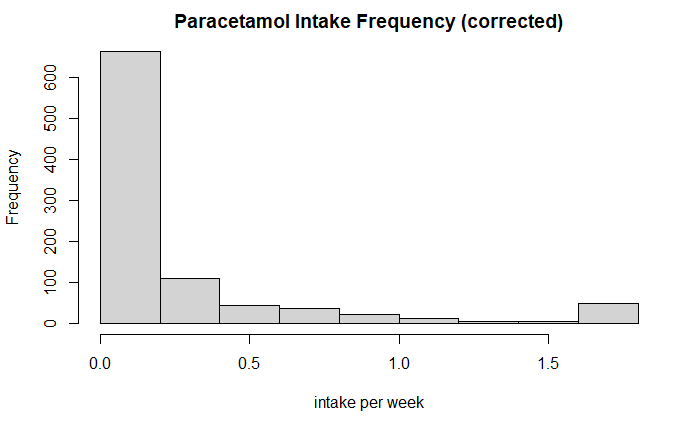


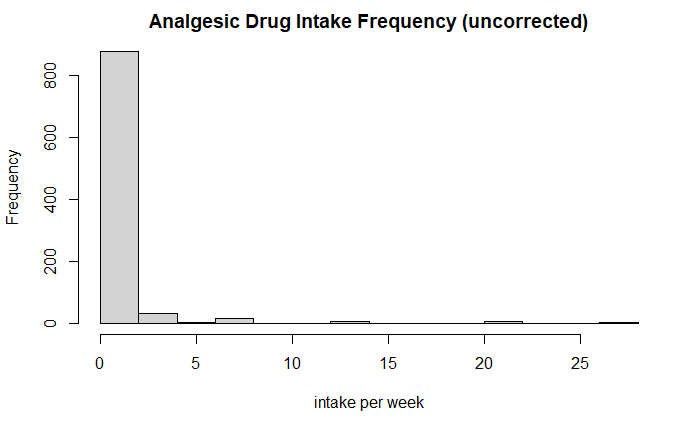

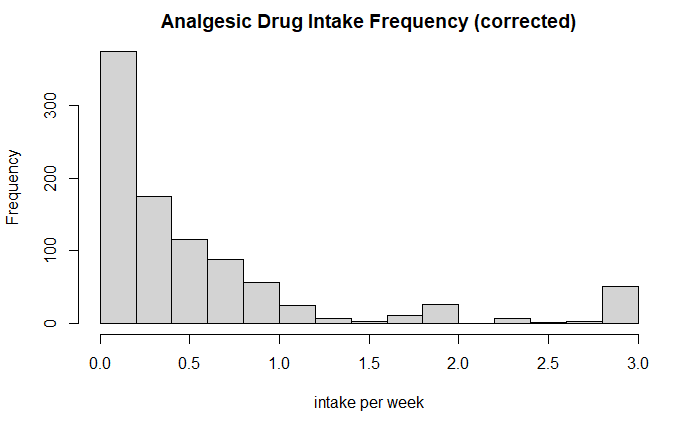

Supplement: Supplementary file 1 — Supplementary Information. [file 41598_2023_45267_MOESM1_ESM.docx]
